# Down-regulation of CCNE1 expression suppresses cell proliferation and sensitizes gastric carcinoma cells to Cisplatin

**DOI:** 10.1042/BSR20190381

**Published:** 2019-06-04

**Authors:** Chao Zhang, Qiang Zhu, Jianzhong Gu, Shan Chen, Qian Li, Liping Ying

**Affiliations:** 1 Rehabilitation Treatment Room of Acupuncture and Moxibustion Department, Zhejiang Provincial Hospital of TCM, Hangzhou, China; 2 Department of Infectious Disease, Zhejiang Provincial Hospital of TCM, Hangzhou, China; 3 Department of Oncology, Zhejiang Provincial Hospital of TCM, Hangzhou, China; 4 Department of Acupuncture and Moxibustion, Zhejiang Provincial Hospital of TCM, Hangzhou, China; 5 Operating Room, Zhejiang Provincial Hospital of TCM, Hangzhou, China

**Keywords:** CCNE1, Cisplatin, gastric carcinoma, proliferation, sensitize

## Abstract

A novel oncogene CCNE1 (cyclin E) is considered to be associated with the development of various tumor types, its role in gastric carcinoma (GC) is little studied and the effect of CCNE1 on chemotherapy also remains unclear. We recruited 55 cases of GC tissues and corresponding normal tissues. Immunohistochemistry (IHC), quantitative real-time PCR (qRT-PCR) and Western blot analysis were performed to detect the expression of CCNE1. We also examined the expression of CCNE1 in gastric mucosal GES-1 cells and five GC cell lines. Silencing CCNE1 was used to assess its effect on proliferation and cell cycle in MGC-803 and NCI-N87 cells, as performed by Cell counting kit-8 (CCK-8) and flow cytometry assay. Meanwhile, cell cycle related genes were also detected through qRT-PCR and Western blot. The results showed CCNE1 up-regulation mainly expressed in GC tissues and GC cell lines, also was associated with tumor node metastasis (TNM) stage and lymphatic invasion. Three-year survival curve analysis showed CCNE1 with high expression had a poor prognosis. Silencing CCNE1 significantly reduced cell viability in 48 h, cultured and arrested cell cycle in G_1_ phase, moreover, Cyclin A, D1 and C-myc all revealed down-regulation in both MGC-803 and NCI-N87 cells. CCNE1 expression was significantly increased at low and moderate concentrations of Cisplatin. Down-regulation of CCNE1 expression would remarkably promote cell apoptosis induced by Cisplatin, and regulate the rate of Bax/Bcl-2. Down-regulation of CCNE1 expression could inhibit cell proliferation and enhance GC cells sensibility to Cisplatin, possibly involving the regulation of Bcl-2 family.

## Introduction

Gastric carcinoma (GC) is one of the most common malignant tumors of digestive tract that seriously endanger human health. The incidence rate is 10–150/100000. There are approximately 93.14 million new cases per year in the world, ranking second in all malignant tumors [1]. Each year, there are approximately 700000 deaths and the mortality ranks fourth among all tumors [2]. Gastric cancer has obvious regional character, with high incidence in Asian countries such as Japan, South Korea and China, accounting for approximately two-thirds of the global total. In China, the incidence and mortality rate of gastric cancer ranks first three in all kinds of malignant tumors [1]. Chemotherapy is the main treatment choice for patients with advanced gastric cancer, among which Cisplatin is the most important and basic first-line treatment drug for gastric cancer [3,4]. The United States approved Cisplatin as a clinical therapy for malignant tumors for the first time in 1978 [5]. At present, Cisplatin is widely used in the treatment of various malignant tumors, including head and neck cancer, ovarian cancer, testicular cancer, bladder cancer, liver cancer, lung cancer and colorectal cancer [6–9]. Patients treated with Cisplatin usually get better results in the initial stage of Cisplatin chemotherapy, but the tumor cells tend to develop resistance to Cisplatin, which seriously restricts the clinical application of Cisplatin [10].

CCNE1 (cyclin E), consisting of four exons and three introns, can transcribe 2.2 kb mRNA with a short half-life of approximately 30 min [11]. The one-third segment of CCNE1 contains a highly conserved region containing approximately 87 amino acids, commonly referred to as cyclin box, which is necessary for cyclin-dependent kinases (CDKs) binding. There are residues rich in proline (P), glutamate (E), aspartic acid (S), threonine (T) in the C-terminal of the CCNE1 protein, called PEST sequence, which plays an important role in protein degradation [12]. In addition, the CCNE1 can be degraded by the Ubiquitin pathway in SKP2 (F-box protein) of Skpl-Cullin-F-box protein (SCF) [13]. CCNE1 is a positive regulator of cell cycle regulation, which promotes G_1_/S phase transition by binding to CDK2 and activating CDK2.

Abnormal overexpression of CCNE1 sustainably activates CDK2, which leads to phosphorylation of substrate pRb, which leads to abnormal cell proliferation [14]. In many malignant tumors, including breast cancer, non-small cell lung cancer and leukemia, the expression of CCNE1 is out of step with the cell cycle, and the expression level is significantly higher than the physiological level [15]. CCNE1 is considered to be an oncogene and is located on the chromosome 19q12 [16], and amplification at 19q12 has been observed in multiple tumor types including gastric cancer [17]. The relationship between CCNE1 expression and prognosis in gastric cancer is still controversial [18,19]. In the present study, we aim to investigate the relationship between CCNE1 expression and prognosis in patients with gastric cancer, and the effect of CCNE1 on proliferation and chemotherapy *in vitro*.

## Materials and methods

### Patients and tissue samples

A total of 55 cases of GC tissues and adjacent normal tissues were taken from specimens after radical gastrectomy at Zhejiang Provincial Hospital of TCM from May 2011 to June 2014. Tissues were not treated with chemotherapy or radiotherapy before resection and divided into two sets. A portion was stored in 4% formaldehyde solution for pathological diagnosis routinely, and other was frozen through direct immersion into liquid nitrogen immediately and kept at −80°C, then processed for quantitative real-time PCR (qRT-PCR) and Western blotting analysis. Two pathologists evaluated the histological diagnosis and differentiation independently. Comparison of CCNE1 expression in clinicopathological parameters are shown in Table 1. The Ethics Committees of hospital approved the study. Written informed consents had been provided by all patients.

**Table 1:** Comparison of CCNE1 expression in clinicopathological parameters

Clinicopathological parameters	*n* (positive rate)	CCNE1 expression		P-value
		Positive (*n*=35)	Negative (*n*=20)	
Age				0.959
<average	25	16	9	
≥average	30	19	11	
Gender				0.27
Female	17	9	8	
Male	38	26	12	
Tumor size (cm)				0.239
<5	30	17	13	
≥5	25	18	7	
TNM stage*				0.039
I+II	23	11	12	
III+IV	32	24	8	
Lymphatic invasion				0.012
No	21	9	12	
Yes	34	26	8	
Differentiation grade				0.068
High-Moderate	19	9	10	
Low	36	26	10	
Ki-67 expression				0.064
Negative	24	12	12	
Positive	31	23	8	

* TNM, tumor node metastasis. TNM stage: to determine the classification of the scope of tumor lesions during cancer treatment.

### Immunohistochemistry

CCNE1 protein expression was located by immunohistochemistry (IHC) using streptavidin-peroxidase (SP) staining. Sections were deparaffinized in xylene, dehydrated with gradient ethanol, and endogenous peroxidase was blocked with 3% H_2_O_2_. Hot of sodium chloride citrate buffer was used to renovate antigen for 20 min. Then samples were incubated with CCNE1 antibody (ab3927, 1:100, Abcam) at 4°C overnight. Samples were washed by PBS and incubated at room temperature for 30 min with secondary antibody HRP–conjugated goat anti-Rabbit IgG (Proteintech, U.S.A.). Diaminobenzidine (DBA) was used as chromogen and Hematoxylin was used to redye. The substitution of PBS for primary antibody was used as a negative control (NC). Selective representative slices were evaluated for staining pattern. IHC was scored based on the tissue positive ratio and intensity, with staining intensity categorized as negative (0), popcorn (1), brown yellow (2), nigger-brown (3), and tissue positive ratio as 0 for negative, 1 for <1/3, 2 for 1/3–2/3 and 3 for >2/3. The immunostains were evaluated by two independent experienced pathologists. The final CCNE1 staining score was obtained by addition tissue positive ratio and intensity rank number, and was defined as follows: staining score of 0 was negative, 2–3 was weakly positive (+), 4 was medium positive (++), 5–6 was strongly positive (+++).

### Cell culture

Normal human gastric mucosal cells (GES-1 cells) and five GC cell lines (SNU-5, KATO III, NCI-N87, AGS, MGC-803) were obtained from ShangHai YIXUAN Biological science and Technology Ltd (Shanghai, China). Cells were cultured in DMEM containing 10% fetal bovine serum, 100 μg/ml penicillin and 100 μg/ml streptomycin, placed in 37°C and 5% CO_2_ incubator. When confluence was close to 75–90%, cells were digested and subcultured.

### qRT-PCR

Total RNA was extracted from tissues or cells by using TRIzol reagent (Invitrogen, U.S.A.) according to the manufacturer’s protocol. CCNE1, cell cycle related RNA (Cyclin A, Cyclin D1 and C-myc), apoptosis related RNA (Bax and Bcl-2) were detected by qRT-PCR in different groups. Reverse transcription was performed with OrimeScript™ RT reagent kit (TaKaRa, Otsu, Shiga, Japan), CCNE1 was at 42°C for 15 min and 85°C for 5 s, cell cycle related and apoptosis related RNAs were at 37°C for 60 min and 95°C for 3 min. The qRT-PCR was performed by SYBR Fast qPCR Mix (Invitrogen, U.S.A.) and primer sequences are summarized in Table 2. Samples were run using the following cycling parameters: CCNE1, 95°C for 1 min, 95°C for 10 s, 60°C for 40 s followed by 40 cycles of 72°C for 30 s and 72°C for 10 min; Cyclin A, 95°C for 5 min, 94°C for 45 s, 53°C for 45 s followed by 35 cycles of 72°C for 45 s and 72°C for 8 min; Cyclin D1, 95°C for 5 min, 94°C for 45 s, 52°C for 45 s followed by 35 cycles of 72°C for 45 s and 72°C for 8 min; C-myc, 94°C for 2 min, 94°C for 30 s, 54°C for 30 s followed by 30 cycles of 72°C for 2 min and 72°C for 6 min; Bax and Bcl-2, 95°C for 30 s, 95°C for 5 s, 60°C for 34 s followed by 40 cycles of 72°C for 2 min and 72°C for 8 min; GAPDH, 95°C for 3 min, 94°C for 40 s, 54°C for 40 s followed by 30 cycles of 72°C for 60 s and 72°C for 3 min. Above primers were purchased commercially (Invitrogen, U.S.A.). Amplified products were electrophoresed through 2% agarose gels. The amount of RNA was calculated using the 2^−ΔΔ*C*^_T_ method.

**Table 2:** Primers used in qRT-PCR

Gene	Primer	Sequence
*CCNE1*	Forward	5′-TACACCAGCCACCTCCAGACAC-3′
	Reverse	5′-CCTCCACAGCTTCAAGCTTTTG-3′
*Cyclin A*	Forward	5′-AGGCTAACCCCACTCTATGAATC-3′
	Reverse	5′-TCTTGCCTTTGGTGGACTA-3′
*Cyclin D1*	Forward	5′-AAAGGAAGCAAGAACCCAT-3′
	Reverse	5′- GTCCGAGATTATCATTACCC-3′
*C-myc*	Forward	5′-GTGGAGTTCAAGCAGGAGAT-3′
	Reverse	5′-CAGAAAGGGATGGAAAGTAG-3′
*Bax*	Forward	5′-TTGCTACAGGGTTTCATCCAG -3′
	Reverse	5′-ATGTTGTTGTCCAGTTCATCG -3′
*Bcl-2*	Forward	5′-GGTGGACAACATCGCTCTG-3′
	Reverse	5′-AGACAGCCAGGAGAAATCAAAC-3′
*GAPDH*	Forward	5′-AACTCCATCATGAAGTGTGA-3′
	Reverse	5′-ACTCCTGCTTGCTGATCCAC-3′

### Western blotting analysis

Total protein was extracted from tissues or cells by use of lysis buffer. Bradford method was used to determine the concentration of proteins. Aliquots supernatant proteins were added with loading buffer and subjected to 10% SDS/PAGE. The resolved proteins were transferred to PVDF membranes (Beyotime, Shanghai, China) and 5% milk with 0.1% Triton X-100 blocked the membranes, and then samples incubated with different primary antibodies: rabbit anti-CCNE1 antibody (ab3927, 1:1000, Abcam, U.S.A.), anti-Cyclin A antibody (sc-53228, 1:1000, Santa Cruz, U.S.A.), anti-Cyclin D1 antibody (ab134175, 1:10000, Abcam, U.S.A.), anti-C-myc antibody (ab32072, 1:1000, Abcam, U.S.A.), anti-Bax antibody (ab182734, 1:1000, Abcam, U.S.A.), anti-Bcl-2 antibody (ab182858, 1:2000, Abcam, U.S.A.) and anti-GAPDH (ab8245, 1:2000, Abcam, U.S.A.) for overnight at 4°C. Blots were then incubated with HRP–conjugated goat anti-Rabbit IgG (Proteintech, U.S.A.) as secondary antibodies. Membranes were washed with TBS three times for 5 min and the blots were viewed with ECL (Thermo Fisher Scientific, Inc., U.S.A.). The quantitation of the relative expression of protein was performed using Quantity one (Bio-Rad, U.S.A.).

### Cell transfection

MGC-803 and NCI-N87 cells were seeded in six-well plates (1.0 × 10^5^) for 24 h before transfection. Silent CCNE1 and empty control plasmids were purchased from Invitrogen (SanMateo, CA, U.S.A.). Transient transfection was operated by Lipofectamine 2000 (Invitrogen, U.S.A.) according to manufacturer’s protocol. A total of siRNA, NC and Lipofectamine 2000 were added to Opti-MEM medium and incubated at 25°C for 10 min, respectively. Then Lipofectamine 2000 was mixed into each well cultured in Opti-MEM RPMI 1640 medium. After 6 h of culturing, the fluid was changed back to RPMI 1640 medium containing 10% FBS.

### Cell counting kit-8

MGC-803 and NCI-N87 cells were plated into 96-well plates at a seeding density of 1 × 10^4^ cells per well for 24 h. After siCCNE1/NC transfected in cells and cells cultured for 24 or 48 h, 10 μl cell counting kit-8 (CCK-8) solution was added in each well and incubated for another 3 h at 37°C. Cell viability was determined by recording the OD at a test wavelength 450 nm using a microplate reader (Thermo Fisher, Massachusetts, U.S.A.).

### Flow cytometry

Cell cycle and apoptosis were detected by flow cytometry. Both the cells were washed twice by PBS fixed in ethanol at 4°C for 30 min, 1000 rpm centrifugation for 5 min. Cells were washed and resuspended in PBS with RNase and propidium iodide (PI, Mlbio, Shanghai, China) at 37°C for 30 min. Apoptosis assay revealed that the cells were washed twice using washing buffer, and the suspension was cultured with Annexin V-PE and PI in the dark at 25°C for 20 min. Binding buffer was needed to be added to each well. And the samples were analyzed by flow cytometry within 1 h.

### Statistical analysis

Statistical analysis was detected by Prism GraphPad version 6.0 software. All data are presented as mean ± standard deviation (SD). Differences were performed using one-way analysis of variance (ANOVA) or χ^2^ test following Tukey’s multiple comparison. The expression of CCNE1 in GC tissues and adjacent tissues of 55 patients were analyzed by paired *t* test. Survival rate were calculated by the Kaplan–Meier method and compared using the log-rank test. A *P*<0.05 was considered significant.

## Results

### Protein expression of CCNE1 and the effect of clinicopathological parameters in patients with GC

**Figure 1:**
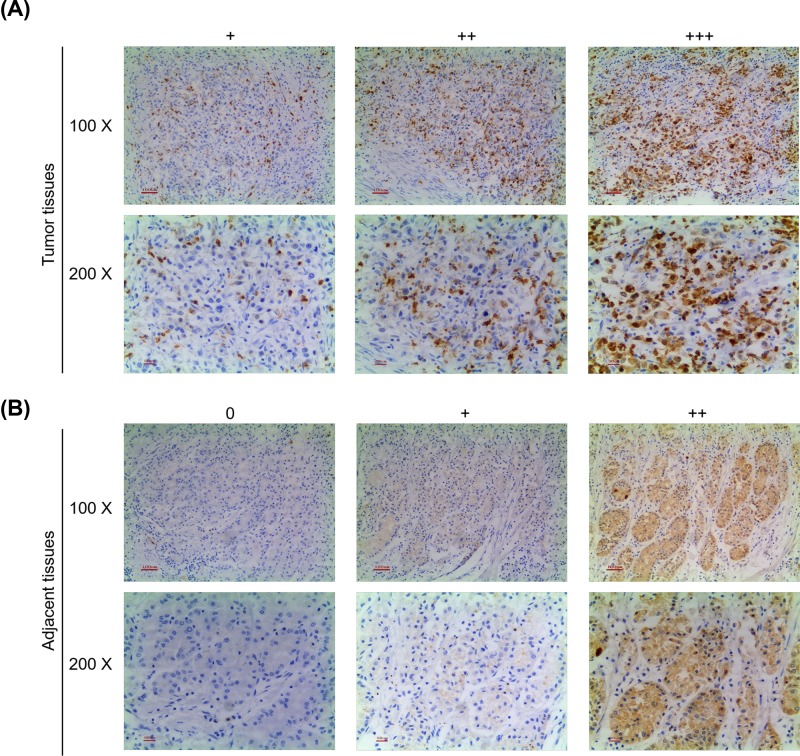
Protein expression of CCNE1 in patients with GC and adjacent tissues (**A**) Representative immunohistochemical staining for CCNE1 from GC tumor tissues and observation using an inverted microscope. (**B**) Representative immunohistochemical staining for CCNE1 from adjacent tissues and observation using an inverted microscope. Both upper (100×), both lower (200×).

Immunohistochemical staining was detected to evaluate protein expression in patients. As shown in Figure 1, both tumor (Figure 1A) and adjacent (Figure 1B) tissues exihibited CCNE1 expression using the final staining score and only normal tissues did not have the expression of CCNE1. Obviously, the immunostaining intensity of CCNE1 in tumor tissues were higher than that in adjacent tissues. Comparison of CCNE1 expression in clinicopathological parameters showed that CCNE1 was significantly associated with Tumor Node Metastasis (TNM) stage and lymphatic invasion (*P*<0.05); however, other clinicopathological parameters like age, gender, tumor size, differentiation grade and Ki-67 expression had no clear correlation with CCNE1 (*P*>0.05, Table 1).

### CCNE1 expression in GC tumor and adjacent tissues and relationship with survival rate

**Figure 2:**
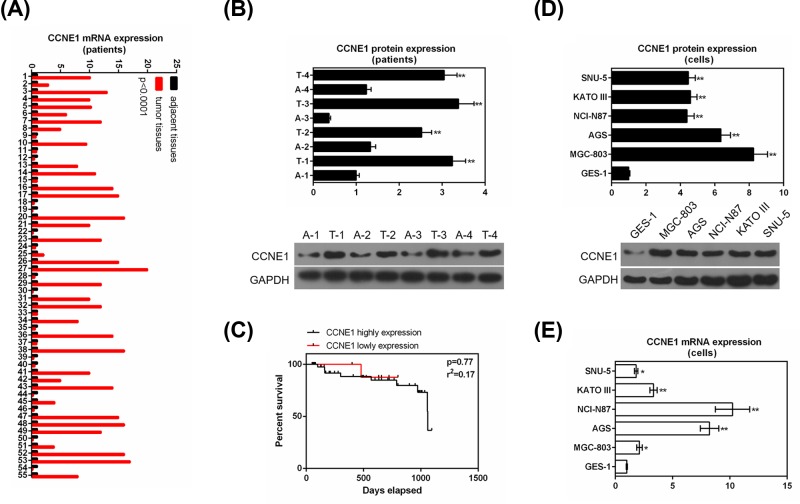
Expression of CCNE1 in tumor, adjacent tissues and GC cell lines, normal gastric mucosal cells and its relationship with survival of GC patients (**A**) Fifty-five patients revealed the relative mRNA levels of CCNE1 in GC tissues and adjacent tissues by qRT-PCR. (**B**) CCNE1 protein levels were evaluated in typical four groups GC tissues as compared with paired adjacent tissues by Western blot (*compared with adjacent tissues, *P<0.05, **P<0.01). (**C**) Three-year survival rate analyses of low and high expressions of CCNE1 by Kaplan–Meier curve. (**D**) Protein levels of CCNE1 in gastric mucosal cells (GES-1) and GC cell lines (SNU-5, KATO III, NCI-N87, AGS, MGC-803) using Western blot (*compared with gastric mucosal cells, *P<0.05, **P<0.01). (**E**) mRNA levels of CCNE1 in normal gastric mucosal cells and GC cell lines by qRT-PCR. GAPDH served as the internal control (*compared with gastric mucosal cells, *P<0.05, **P<0.01). Data were expressed as mean ± SD from three independent experiments.

Fifty-five patients were recruited in the present study, and the expression of CCNE1 in GC tissues and adjacent tissues were detected by qRT-PCR (Figure 2A) and representative four pairs of tumor and adjacent tissues were detected by Western blot (Figure 2B). The result showed that tumor tissues had a significantly higher mRNA expression than adjacent tissues (*P*<0.0001, Figure 2A) and the up-regulation expression of CCNE1 relative to the GC tissue was found in 35 cases, what was more, protein expression of CCNE1 had similar results with RNA (*P*<0.01, Figure 2B). To understand the effect of CCNE1 expression on prognostic, we assessed the relationship between CCNE1 expression and 3-year survival analysis. We found that high expression of CCNE1 had a poor 3-year survival rate, though it had no clear effect (*P*=0.77, Figure 2C).

### CCNE1 expression in normal gastric mucosal cells and GC cell lines

The expression of CCNE1 in normal gastric mucosal cells and GC cell lines were determined. The result showed that GC cells completely high protein expression of CCNE1 compared with normal gastric mucosal cells (*P*<0.01, Figure 2D). The mRNA level also showed similar results that all four cell lines SNU-5 (*P*<0.05), KATO III (*P*<0.01), NCI-N87 (*P*<0.01), AGS (*P*<0.01), MGC-803 (*P*<0.05) had significantly high expression vs normal cells (Figure 2E). We selected MGC-803 and NCI-N87 cells to be the subject of the following experiments.

### The expression of CCNE1 in silent CCNE1 GC cells

**Figure 3:**
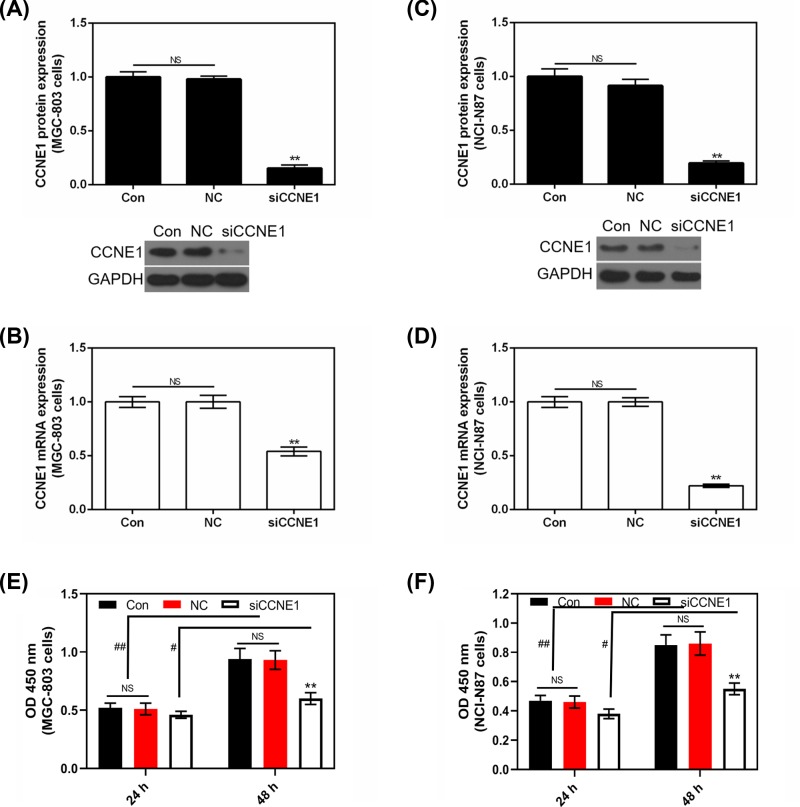
Transfection efficiency of CCNE1 and the viability effect of siCCNE1 on GC cells (**A**) The effect of silencing CCNE1 on protein level of CCNE1 in MGC-803 cell. (**B**) The effect of silencing CCNE1 on CCNE1 mRNA expression in MGC-803 cell. (**C**) The effect of silencing CCNE1 on protein level of CCNE1 in NCI-N87 cell. (**D**) The effect of silencing CCNE1 on CCNE1 mRNA expression in NCI-N87 cell. (**E**) After 24 or 48 h of culture, the effect of silencing CCNE1 on cell viability in MGC-803 cell. (**F**) After 24 or 48 h of culture, the effect of silencing CCNE1 on cell viability in NCI-N87 cell. GAPDH served as an internal control. Data were expressed as mean ± SD from three independent experiments (*compared with control, **P<0.01; ^#^as indicated, ^#^*P*<0.05, ^##^*P*<0.01).

To detect the transfection efficiency of CCNE1 in MGC-803 and NCI-N87 cells, both protein and mRNA levels were assessed. Control and NC had no obvious difference in MGC-803 (Figure 3A,B), NCI-N87 (Figure 3C,D) cells and both in protein and mRNA levels. Silencing CCNE1 completely decreased the expression of CCNE1 in two GC cells (*P*<0.01).

### Silencing CCNE1 reduces GC cell viability after 48 h of culturing

As Figure 3E,F shows, the cell viability was lower after 24 h of culturing than that after 48 h, and silencing CCNE1 did not have significant effect on cell viability after 24 h of culturing compared with Control or NC (*P*>0.05). Whereas, after 48 h of culturing, silencing CCNE1 sharply reduced cell viability both in MGC-803 and NCI-N87 cells compared with control (*P*<0.01, Figure 3E,F).

### Silencing CCNE1 arrests cell cycle in GC cells

**Figure 4:**
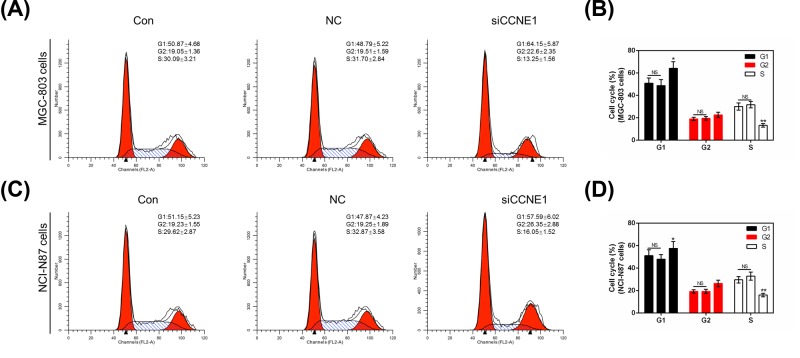
The effect of silencing CCNE1 in cell cycle in GC cells (**A**) Cell cycle was detected by flow cytometry in MGC-803 cells. (**B**) The rate of MGC-803 cell cycle was shown as bar diagrams. (**C**) Cell cycle was detected by flow cytometry in NCI-N87 cells. (**D**) The rate of NCI-N87 cell cycle was shown as bar diagrams. Data were expressed as mean ± SD from three independent experiments (*compared with control, *P<0.05, **P<0.01).

**Figure 5:**
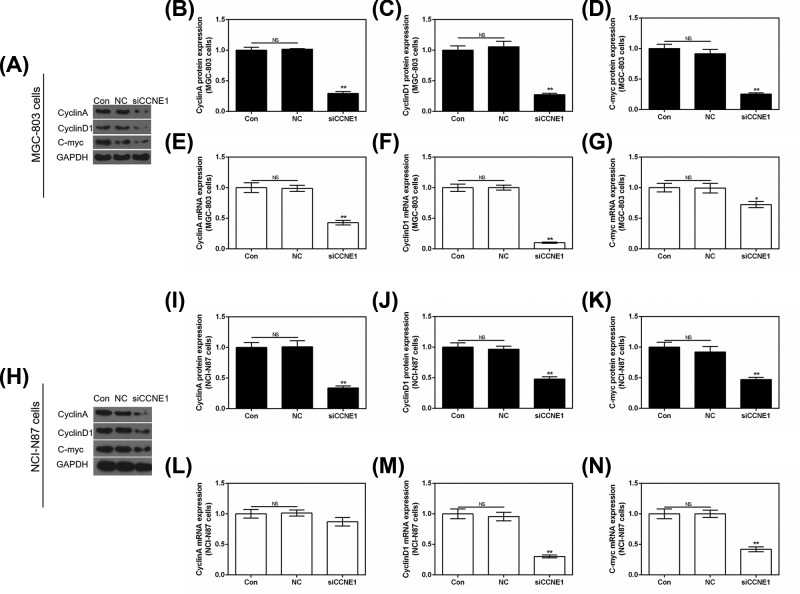
The effect of silencing CCNE1 in cell cycle related genes in GC cells (**A**) Protein expression of Cyclin A, Cyclin D1 and C-myc were assessed using Western blot in MGC-803 cells. Cyclin A (**B**), Cyclin D1 (**C**) and C-myc (**D**) protein expressions shown as bar diagrams in MGC-803 cells. The mRNA level of Cyclin A (**E**), Cyclin D1 (**F**) and C-myc (**G**) were assessed by qRT-PCR in MGC-803 cells. (**H**) Protein expression of Cyclin A, Cyclin D1 and C-myc were assessed using Western blot in NCI-N87 cells. Cyclin A (**I**), Cyclin D1 (**J**) and C-myc (**K**) protein expressions shown as bar diagrams in NCI-N87 cells. The mRNA level of Cyclin A (**L**), Cyclin D1 (**M**) and C-myc (**N**) were assessed by qRT-PCR in NCI-N87 cells. GAPDH served as the internal control. Data were expressed as mean ± SD from three independent experiments (*compared with control, *P<0.05, **P<0.01).

The cell cycle was detected by flow cytometry. In MGC-803 cells, we found silencing CCNE1 could significantly up-regulate (*P*<0.05, Figure 4B) the rate of G_1_ phase from 50.87 ± 4.68 or 48.79 ± 5.22 to 64.15 ± 5.87 (Figure 4A) and markedly decrease the rate of S phase from 30.09 ± 3.21 or 31.70 ± 2.84 to 13.25 ± 1.56 compared with control or NC. The rate of G_2_ phase had no remarkable difference with control (*P*>0.05). In NCI-N87 cells revealed the similar results that silencing CCNE1 could arrest cell cycle in G_1_ phase (Figure 4C,D). Cell cycle related genes Cyclin A, Cyclin D1 and C-myc were also evaluated by use of Western blot and qRT-PCR. In MGC-803 cells, compared with control or NC, silencing CCNE1 completely attenuated the mRNA expression of Cyclin A (*P*<0.01, Figure 5E), Cyclin D1 (*P*<0.01, Figure 5F) and C-myc (*P*<0.05, Figure 5G), and obviously decreased the three protein levels (*P*<0.01, Figure 5A–D). In silent CCNE1 NCI-N87 cells, both protein and mRNA level of three genes showed similar results (*P*<0.01, Figure 5H–K,M,N) with MGC-803 cells, only Cyclin A mRNA level showed a difference that silencing CCNE1 reduced its expression inconspicuously (*P*>0.05, Figure 5L).

### The effect of Cisplatin on the expression of CCNE1 in GC cells

**Figure 6:**
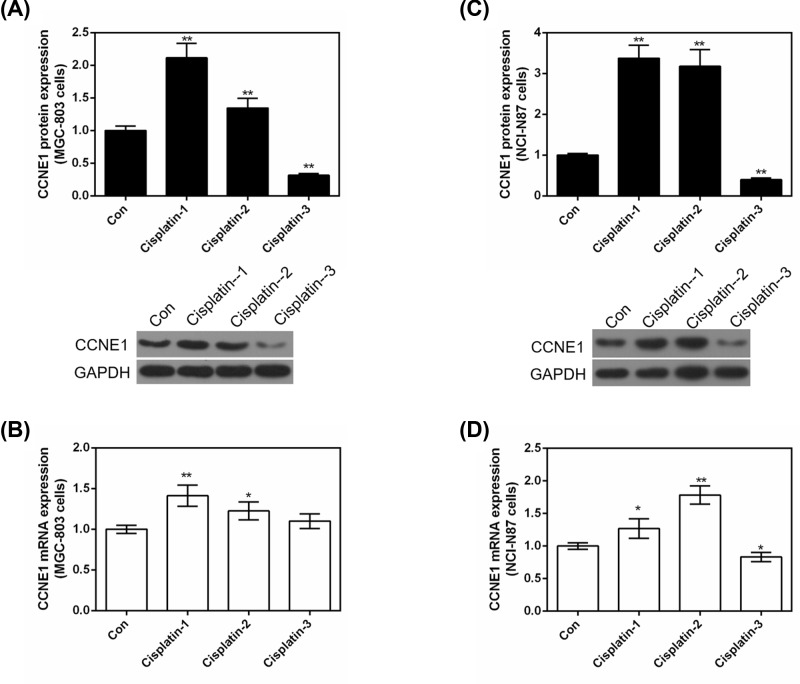
The effect of Cisplatin on the expression of CCNE1 in GC cells Treated MGC-803 or NCI-N87 cells with different concentrations (2, 8, 16 μg/ml) of Cisplatin. (**A**) The protein level of CCNE1 was detected by Western blot in MGC-803 cells. (**B**) mRNA level of CCNE1 was assessed by qRT-PCR in MGC-803 cells. (**C**) The protein level of CCNE1 was detected by Western blot in NCI-N87 cells. (**D**) mRNA level of CCNE1 was assessed by qRT-PCR in NCI-N87 cells. GAPDH served as an internal control. Data were expressed as mean ± SD from three independent experiments (*compared with control, *P<0.05, **P<0.01).

As Figure 6 shows, we detected the effect of different concentrations of Cisplatin on the expression of CCNE1 both in protein and mRNA levels. In MGC-803 cells, 2 and 8 μg/ml of Cisplatin significantly increased the protein expression of CCNE1 (*P*<0.01, Figure 6A), but 16 μg/ml of Cisplatin showed a sharp decreasing expression of CCNE1 compared with control (*P*<0.01). mRNA level also showed that low (*P*<0.01, Figure 6B) and medium (*P*<0.05) concentrations of Cisplatin promoted the expression of CCNE1, high concentration of Cisplatin had no comparison with control (*P*>0.05). And this phenomenon could be explained: (i) high concentration of cisplatin may decrease CCNE1 protein expression directly; (ii) high concentration of cisplatin may affect other genes which could also affect CCNE1 protein expression. The effect of Cisplatin on the expression of CCNE1 in NCI-N87 revealed similar result (Figure 6C,D) with MGC-803 cells. The increased expression of CCNE1 with low and medium concentrations of Cisplatin treatment indicated that CCNE1 might produce resistance to these concentrations of Cisplatin.

### Down-regulation of CCNE1 expression enhances Cisplatin-induced cell apoptosis in GC cells

**Figure 7:**
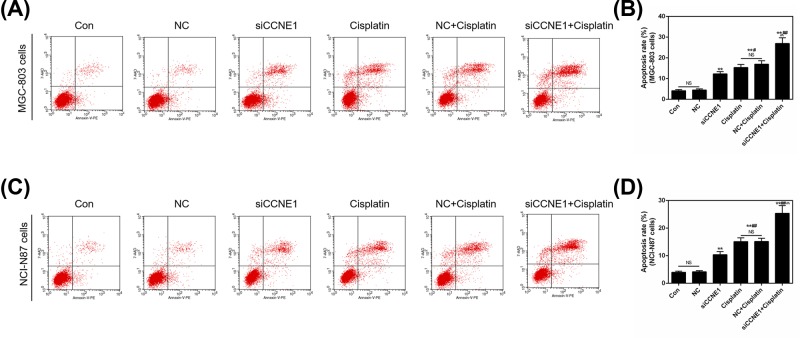
The effect of silencing CCNE1 and Cisplatin on cell apoptosis in GC cells Cells were divided into six groups, including control, NC, siCCNE1, Cisplatin (8 μg/ml), NC+Cisplatin (8 μg/ml) and siCCNE1 + Cisplatin (8 μg/ml). (**A**) The apoptosis was detected by flow cytometry in MGC-803 cells. (**B**) Apoptosis rate was shown as bar diagrams in MGC-803 cells. (**C**) The apoptosis was detected by flow cytometry in NCI-N87 cells. (**D**) Apoptosis rate was shown as bar diagrams in NCI-N87 cells. Data were expressed as mean ± SD from three independent experiments (*compared with control, ^#^compared with siCCNE1, ^∧^ compared with Cisplatin, ^#^*P*<0.05, ^**^/^##^/^∧∧^*P*<0.01).

**Figure 8:**
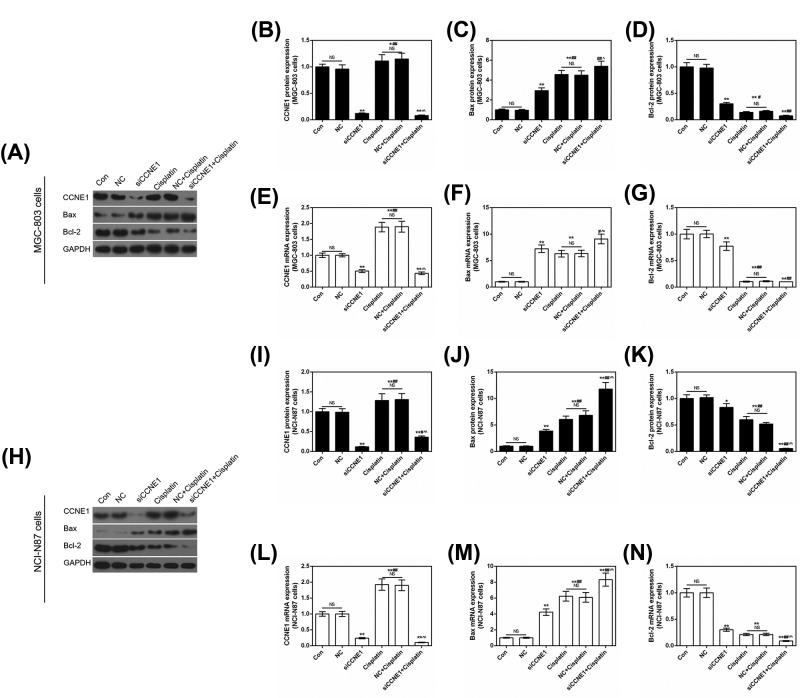
The effect of silencing CCNE1 and Cisplatin on the expression of apoptosis related genes and CCNE1 (**A**) Protein expression of CCNE1, Bax and Bcl-2 were assessed using Western blot in MGC-803 cells. CCNE1 (**B**), Bax (**C**) and Bcl-2 (**D**) protein expressions are shown as bar diagrams in MGC-803 cells. The mRNA level of CCNE1 (**E**), Bax (**F**) and Bcl-2 (**G**) were assessed by qRT-PCR in MGC-803 cells. (**H**) Protein expression of CCNE1, Bax and Bcl-2 were assessed using Western blot in NCI-N87 cells. CCNE1 (**I**), Bax (**J**) and Bcl-2 (**K**) protein expressions showed as bar diagrams. The mRNA level of CCNE1 (**L**), Bax (**M**) and Bcl-2 (**N**) were assessed by qRT-PCR in NCI-N87 cells. GAPDH served as an internal control. Data were expressed as mean ± SD from three independent experiments (*compared with control, ^#^compared with siCCNE1, ^∧^ compared with Cisplatin, */^#^/^∧^*P*<0.05, **/^##^/^∧∧^*P*<0.01).

The former result showed that medium concentration of Cisplatin rendered the high expression of CCNE1, therefore, we applied silent CCNE1 to study its effect on Cisplatin in GC cells. The apoptosis assay was determined using flow cytometry. The result showed that both silencing CCNE1 and 8 μg/ml Cisplatin could significantly increase apoptosis rate in MGC-803 and NCI-N87 cells compared with control or NC (*P*<0.01, Figure 7A–D), Cisplatin promoted apoptosis stronger than siCCNE1 (*P*<0.05, Figure 7B; *P*<0.01, Figure 7D). Surprisingly, silencing CCNE1 pronouncedly enhanced Cisplatin-induced cell apoptosis (*P*<0.01) in two GC cells. And this result could support above result that increased expression of CCNE1 indicated that CCNE1 might produce resistance to cisplatin. The expressions of related genes were also detected by Western blot and qRT-PCR. As shown by Figure 8A,B,E,H,I,L, addition of siCCNE1, the expression of CCNE1 would significantly down-regulate compared with control or NC in two GC cells (*P*<0.01). Meanwhile, the decreasing effect of siCCNE1 was significantly higher than that of Cisplatin increasing, and represented down-regulation expression of CCNE1 finally (*P*<0.01). The pro-apoptosis gene *Bax* showed single siCCNE1 or Cisplatin significantly increased the protein and mRNA levels of Bax in MGC-803 and NCI-N87 cells (*P*<0.01, Figure 8A,C,F,H,J,M). When siCCNE1 combined with Cisplatin, the increasing expression of Bax was more pronounced than that of single effect. The combination is a synergistic effect. The anti-apoptosis Bcl-2 showed opposite phenomenon that siCCNE1 and Cisplatin could significantly decrease the expression of Bcl-2 (*P*<0.01, Figure 8A,D,G,H,K,N). Also, siCCNE1 + Cisplatin could significantly down-regulate the expression of Bcl-2 compared with siCCNE1 in MGC-803 cells (*P*<0.01, Figure 8D,G) or Cisplatin in NCI-N87 cells (*P*<0.01, Figure 8K,N).

## Discussion

CCNE1 is closely related to the occurrence and development of many tumors such as epithelial ovarian cancer, inflammatory breast cancer, non-muscle invasive bladder cancer, colorectal cancer, ovarian clear cell carcinoma, hepatomegaly, osteosarcoma, glioma [20–27]. In this work, we assessed the expression of a novel oncogenic gene *CCNE1* in GC patients and their adjacent normal tissues using IHC, qRT-PCR and Western blot analysis. Our results demonstrated that CCNE1 was mainly highly expressed in gastric cancer tissues and the clinicopathological characteristics showed that it was closely associated with TNM stage and lymphatic invasion. Cell experiments in protein and RNA level also confirmed that CCNE1 had higher expression in five GC cells than that in gastric mucosal cells. What was more, CCNE1 might play an independent prognostic factor that high expression of CCNE1 had a poor 3-year survival in GC patients. Various previously published literatures on this topic revealed that high CCNE1 expression in GC had a poor prognosis [19,28–37]. However, the findings of Takano et al.’s study [18] do not support the above view, they believe that the prognosis of patients with CCNE1 positive expression of gastric cancer was better than that of negative expression and it was speculated that this may be related to the inactivation of CCNE1 (CyclinE)/CDK2 complexes. Therefore, the relationship between CCNE1 expression and prognosis in gastric cancer is still controversial, and we need to expand the number of cases in future experiments.

In addition, we detected the role of CCNE1 in two GC cells through silencing CCNE1. Both in MGC-803 and NCI-N87 cells, silencing CCNE1 could significantly inhibit cell proliferation in 48 h culture, arrest cell cycle in G_1_ phase. Moreover, siCCNE1 remarkably decreased the expression of cell cycle related genes Cyclin A, Cyclin D1 and C-myc. As we all know they all act as important cell cycle regulators, Cyclin A is involved in both G_1_/S and G_2_/M transitions, which is not only the step of G_1_ to S phase limit, but also the promotion transition of G_2_ to M phase. When cyclin A and cyclin E are overexpressed, the regulation of Rb factor will be abnormal, leading to uncontrolled growth of cells [38,39]. Cyclin D1 binds to CDK 4/6 (CDK4/CDK6) and forms a complex that drives cells from the G_1_ phase to the S phase, promoting cell proliferation [40,41]. C-myc regulates the key points of G_1_ phase at multiple levels, promotes the formation of cyclin E-CDK2 into active free state, and is activated by the cyclin active kinase CAK, which leads to the release of E2F, and finally allows cells to enter the S phase from G_1_ [42,43].

To investigate the effect of CCNE1 on chemotherapy *in vitro*, we used silencing CCNE1 to test its function in chemotherapy sensitivity of Cisplatin in gastric cancer cell lines. CCNE1 expression was significantly increased at low and moderate concentrations of Cisplatin, suggesting that CCNE1 was resistant to Cisplatin at these concentrations. When siCCNE1 and Cisplatin were used in combination, the expression of CCNE1 showed sharp down-regulation, and Annexin V-PE revealed significant apoptosis induction compared with single siCCNE1 or single Cisplatin treatment. The combination was a synergistic effect. The result indicated that down-regulation of CCNE1 expression could increase apoptosis induced by Cisplatin in gastric cancer cells. Though, 8 μg/ml of Cisplatin could increase the expression of CCNE1 does not mean that Cisplatin (8 μg/ml) definitely lowers apoptosis. Maybe Cisplatin (8 μg/ml) still could affect other pro-apoptosis genes that could induce cell apoptosis. Similarly, Liu et al.’s [44] research also supported ours. And a more in-depth research will be launched in the future study to explain this result clearly. What was more, examination of apoptosis related genes showed that CCNE1 down-regulation significantly increased Bax and attenuated Bcl-2. Bcl-2 gene family is involved in signal transduction pathway of apoptosis and plays an important role in chemotherapy-induced apoptosis [45]. Bcl-2 is an important anti-apoptosis gene with a variety of biological functions. It is not only involved in the inhibition of apoptosis, but is also an independent drug resistance gene. Blocking or down-regulating the expression of Bcl-2 can promote the apoptosis of tumor cells and enhance the sensitivity of tumor cells to radiotherapy and chemotherapy, so as to improve the therapeutic effect of tumors [46,47]. Bax-transduced gastric cancer cells could promote mitochondria release of cytochrome *c*, which in turn activated Caspase 3, thereby enhancing chemotherapeutic drug-induced apoptosis [48].

Certainly, our study still exhibited some limitations to validate CCNE1 might produce resistance to Cisplatin, for example, applying overexpression of CCNE1 in combination to Cisplatin and other experiments including cell viability and cell cycle. We are going to launch a more comprehensive study.

Taken together, the present study demonstrates that CCNE1 plays a key role in GC. Down-regulation of CCNE1 expression suppressed GC cells proliferation, arrested cell cycle in G_1_ phase, additionally, it participated in chemoresistance, by which CCNE1 could regulate Cisplatin-induced apoptosis and CCNE1 down-regulation enhance the sensitivity of GC cells to Cisplatin, possibly involved in the regulation of Bcl-2 family.

## Abbreviations

Bax: Bcl-2 associated X protein
Bcl-2: B cell leukemia/lymphoma 2
CAK: cyclin active kinase
CCNE1: cyclin E
CDK: cyclin-dependent kinase
GAPDH: glyceraldehyde phosphate dehydrogenase
GC: gastric carcinoma
HRP: horseradish peroxidase
IHC: immunohistochemistry
NC: negative control
NS: no significance
OD: optical density
PE: Phycoerythrin
PEST: Acronyms for proline, glutamate, aspartic acid and threonine
PI: propidium iodide
qRT-PCR: quantitative real-time PCR
RPMI1640: Roswell Park Memorial Institute1640
SD: standard deviation
TNM: tumor node metastasis
